# Pregnancy outcome after first-trimester exposure to JAK inhibitors - an evaluation of the German Embryotox cohort

**DOI:** 10.1186/s40360-026-01263-4

**Published:** 2026-10-20

**Authors:** Anna-Sophia Habbel, Evelin Beck, Anne Mueller, Katarina Dathe

**Affiliations:** Charité – University Medical Center Berlin, Corporate Member of Freie Universität Berlin and Humboldt-Universität zu Berlin, Institute of Clinical Pharmacology and Toxicology, Embryotox Center of Clinical Teratology and Drug Safety in Pregnancy, Augustenburger Platz 1, 13353 Berlin, Germany

**Keywords:** Pregnancy [MeSH-terms], Congenital abnormalities [MeSH-terms], Abortion, spontaneous [MeSH-terms], Janus kinase inhibitors [MeSH-terms], Baricitinib, Filgotinib, Tofacitinib, Upadacitinib

## Abstract

**Background:**

Janus kinase inhibitors (JAKi) are increasingly being used in the management of autoimmune diseases across rheumatology, gastroenterology, and dermatology, including in women of childbearing age. Consequently, pregnancies occurring during JAKi treatment are becoming more common. However, uncertainties regarding their safety during pregnancy remain, and current human data are insufficient to allow a comprehensive assessment of fetal risk.

**Methods:**

Observational data of pregnancies with first trimester JAKi exposure, recorded in the German Embryotox database, was analyzed. Pregnancies were prospectively enrolled between January 1, 2017, and November 15, 2025. Study endpoints included major congenital anomalies, pregnancy loss, and neonatal parameters.

**Results:**

The cohort comprised a total of 44 pregnancies exposed to baricitinib (*n* = 17), filgotinib (*n* = 3), tofacitinib (*n* = 8), or upadacitinib (*n* = 16). A total of 32 live births were recorded. Among these, two major congenital anomalies were observed, one ventricular septal defect and one hypospadias. Five spontaneous abortions occurred, including one case of fetal anencephaly, and seven elective terminations of pregnancy were reported. Additional neonatal assessments revealed two preterm births at 36 weeks’ gestation, three infants born small for gestational age, and two newborns large for gestational age.

**Conclusions:**

The current evidence is insufficient to establish or exclude an association between JAK inhibitor exposure and adverse fetal outcomes. To date, the potential for harmful effects cannot be ruled out due to the limited available data. Therefore, routine use of JAKi during pregnancy is not recommended and treatment should be approached with caution, and requires a critical risk-benefit assessment until further data is obtained.

**Trial registration:**

The study was retrospectively registered in the German Clinical Trials Register on March 16, 2026 (DRKS00039622).

## Background

Various autoimmune and inflammatory diseases in dermatology, rheumatology, and gastroenterology are mediated by cytokine dysregulation [1, 2]. These conditions manifest more frequently in women of childbearing age and have been associated with a high degree of morbidity [3]. Therefore, ensuring adequate treatment for these diseases is imperative for the well-being of both mother and child during pregnancy and breastfeeding [3]. In case of severe disease progression one treatment option is the therapy with Janus kinase inhibitors (JAKi). This relatively novel group of substances consists of four subgroups, JAK1, JAK2, JAK3, and tyrosine kinase 2 (TYK2) and very effectively inhibits intracellular signaling pathways in inflammatory diseases [4]. In addition, the Janus kinase signal transducer and activator of transcription (JAK/STAT) pathway regulates DNA transcription and a wide range of cellular functions including proliferation, migration, differentiation, and apoptosis [2, 5–7]. Furthermore, angiogenesis and fetal hematopoiesis are affected by JAK/STAT signaling [8–11].

It was shown that JAKi, as small molecules, cross the placental barrier in humans [12–14]. Therefore, and in light of the limited and heterogeneous human data regarding pregnancy exposure to JAKi, current clinical guidance emphasizes caution [15–18]. Available published data comprise an abstract reporting on a retrospective cohort [19], three small case series [12, 20, 21], individual case reports [14, 22–33] and analyses from manufacturers’ studies and pharmacovigilance databases [34–37], all of which provide only preliminary insights. The results of preclinical data from animal studies have raised concerns [15–18] but the relevance of these findings to human pregnancy remains uncertain.

This evaluation of JAKi-related pregnancy data and outcomes in the Embryotox cohort aims to enhance the knowledge in this area by utilizing data collected independently of industry-funded registries. The primary objective was to assess the incidence of major congenital anomalies and adverse pregnancy outcomes subsequent to JAKi treatment during the first trimester. Neonatal parameters following intrauterine fetal exposure were additionally analyzed.

## Methods

The Berlin Embryotox Center provides a risk counselling service, primarily for medication during family planning, pregnancy, and lactation upon request from health care professionals and patients. All patients were treated on an outpatient basis by their respective physicians and were registered following an inquiry regarding drug counselling in pregnancy submitted to Embryotox. Additionally, Embryotox acts as a national center for assessing and reporting suspected adverse drug reactions during pregnancy.

### Data collection

At the initial counselling contact, a structured questionnaire is used to systematically record all relevant data concerning drug exposure, treatment indications, maternal characteristics (including age, body mass index (BMI) and comorbidities) and relevant obstetric and family history. All patients and health care professionals are informed about data privacy and collection and how data is used, and they provide their consent for the pregnancy follow-up information to be collected.

A request for follow-up data is made eight weeks after the estimated date of birth (EDOB) in predefined constellations by a structured questionnaire. Maternal data include again information regarding drug exposure during pregnancy, pregnancy complications, further diseases including infections and fever, and details of delivery. Further information is requested if pregnancy loss occurred. Data on the child comprise gestational age at birth, sex, birth weight, length, head circumference, Apgar scores, umbilical pH, and any congenital and postnatal abnormalities, or complications within the first few weeks of life. In addition, the pregnancy follow-up questionnaire asks for the results of the initial pediatric standard examinations conducted within the first six weeks of life performed in Germany [38]. A case-by-case plausibility check is performed on a routine basis, and if the data are insufficient or imprecise we attempt to clarify them further. All relevant data are structurally archived in the Embryotox database (Medango provided by PharmApp Solutions GmbH).

### Study design and study cohort

For this observational study, prospectively ascertained pregnancies exposed to JAKi between January 1, 2017, and November 15, 2025 were considered. Cases were designated as “prospective” when the pregnancy outcome was unknown and no prenatal pathology had been diagnosed at the time of initial contact.

**Fig. 1:**
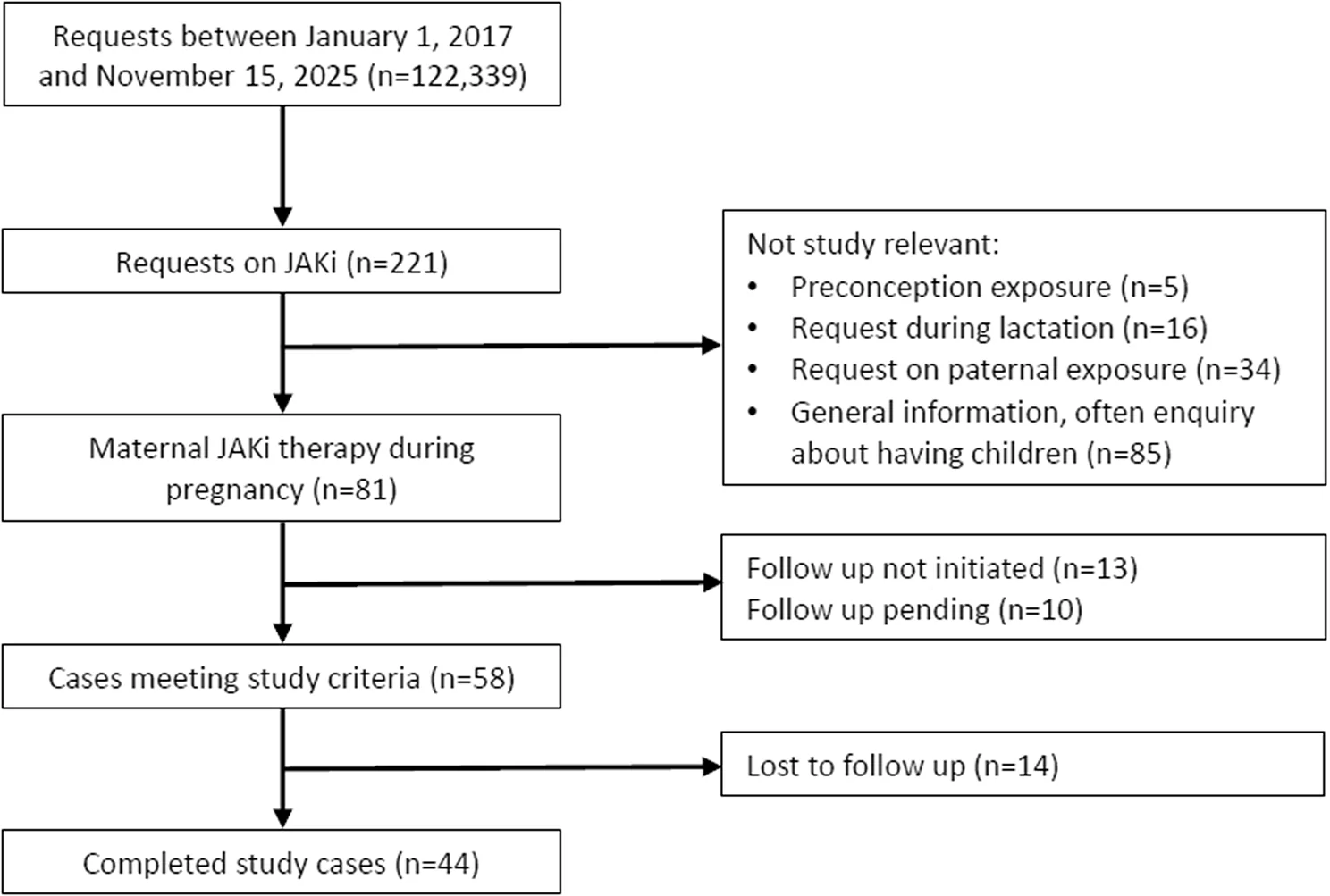
Number of JAK inhibitor (JAKi) exposed pregnancies

Pregnancies were defined as exposed in case of any exposure to JAKi in usual dosage between 2 + 0 weeks after the last menstrual period (LMP) and delivery. First trimester exposure was defined as any exposure between GW 2 + 0 and 12 + 6 after LMP. Treatment might have started earlier and could have lasted longer. The presence of teratogenic or fetotoxic co-medication was not an exclusion criterion but rather an aspect to be given consideration during the assessment. Further criteria for generating the study cohort are shown in Fig. 1. Gestational age was estimated by ultrasound during the early first trimester. If this data was not available, gestational age was calculated from the first day of the LMP. Spontaneous abortion was defined as the unintended pregnancy loss of a fetus weighing less than 500 g or less than 24 completed weeks after LMP, if the weight was unknown. The term ‘small-for-gestational-age’ (SGA) was used to describe an infant with a birth weight less than the 10th percentile for both sex and gestational age, the same applies to ‘large for gestational age’ (LGA) for values above the 90th percentile. Prematurity was defined as delivery at less than 37 completed weeks of gestation.

Birth defects were classified according to the EUROCAT classification system into ‘major’, ‘minor for exclusion’ and ‘genetic’ (no cases were reported in the study cohort) according to guidelines of the European network of population-based registries for the epidemiological surveillance of congenital anomalies (EUROCAT, Guide 1.5) [39].

### Study endpoints and data analysis

Primary study endpoints included the occurrence of major birth defects and pregnancy loss subsequent to exposure to JAKi during the first trimester. Secondary endpoints were adverse pregnancy outcomes encompassing gestational age at delivery (prematurity), birth weight and head circumference. For the maternal and infant characteristics descriptive statistics were applied. For metric values, the median and interquartile range (IQR) are reported. Categorical values are presented as proportions. Calculations and illustrations were generated with R version 4.5.1 [40].

### Participant and public involvement

There was no patient or public involvement in setting the research questions, designing the study or interpretation of study results.

### Funding

This work was supported by the German Federal Institute for Drugs and Medical Devices (BfArM, sub-project of grant number V-2025.3 / 68605 / 2026–2029). The funder had no role in study design, data collection and analysis, decision to publish, or preparation of the manuscript. The study was retrospectively registered in the German Clinical Trials Register on March 16, 2026 (DRKS00039622).

## Results

A total of 211 requests on JAKi were retrieved from the Embryotox database between January 1, 2017 and November 15, 2025. In 44 of the 58 prospective cases that met the study’s criteria, follow-up was completed (Fig. 1). There were no cases that were only exposed after the first trimester.

### Maternal characteristics

The median age of pregnant women was 31 years and the median BMI at the start of pregnancy was 23.5 kg/m^2^. Smoking and alcohol consumption was reported in two (4.9%) and five (12.2%) of pregnancies, respectively. Of the evaluated pregnancies for which this information could be obtained, only six (17.2%) were primarily planned. Table 1 summarizes the relevant maternal characteristics.

**Table 1:** Maternal characteristics of JAKi exposed women (*n* = 44)

**Age, n (%)**	44 (100)
Age, median years (IQR)	31 (29–35)
**BMI, n (%)**	41 (93.2)
BMI, median kg/m^2^ (IQR)	23.5 (20.7–26.8)
**Educational level, n (%)**	27 (61.4)
No leaving exam	0 (0)
9 years exam	1 (3.7)
10/11 years exam	12 (44.4)
Secondary school exam	7 (25.9)
Academic study	7 (25.9)
**Smoking, n (%)**	41 (93.2)
No	39 (95.1)
≤5 cig/day	0 (0)
>5 cig/day	2 (4.9)
**Alcohol, n (%)**	41 (93.2)
No	36 (87.8)
≤1 drink/day	2 (4.9)
>1 drink/day	3 (7.3)
**Social drugs, n (%)**	41 (93.2)
Yes	0 (0)
No	41 (100)
**Pregnancy wanted, n (%)**	34 (77.3)
No	2 (5.9)
Ambivalent	8 (23.5)
Accepted	18 (52.9)
Planned	6 (17.6)
**Previous pregnancies, n (%)**	41 (93.2)
0	16 (39)
1	9 (22)
2	11 (26.8)
3 or more	5 (12.2)
**Previous deliveries, n (%)**	41 (93.2)
0	18 (43.9)
1	13 (31.7)
2	9 (22)
3 or more	1 (2.4)
**Previous miscarriages, n (%)**	38 (86.4)
0	29 (76.3)
1	6 (15.8)
2 or more	3 (7.9)
**Previous elective terminations, n (%)**	41 (93.2)
0	39 (95.1)
1	2 (4.9)
2 or more	0 (0)
**Previous children with birth defect, n (%)**	41 (93.2)
0	39 (95.1)
1	2 (4.9)
2 or more	0 (0)
**Gestational diabetes, n (%)**	33 (75)
Yes	5 (15.2)
No	28 (84.8)
**Pre-eclampsia, n (%)**	32 (72.7)
Yes	0 (0)
No	32 (100)
**Week at first contact, n (%)**	44 (100)
Week at first contact, median week (IQR)	6.5 (4.7–9.7)

For age, BMI and week at first contact, median and interquartile range (IQR) are presented

In the study cohort, two urinary tract infections, as well as one case each of Covid-19, influenza A, Group B Streptococcus colonization, and herpes zoster were recorded. No other infection covered by the routine infection screening offered to all pregnant women in Germany was reported.

An overview of the underlying medical conditions of the pregnant women treated is given in Table 2, encompassing rheumatological (*n* = 30), gastroenterological (*n* = 10), and dermatological (*n* = 4) diagnoses. Due to the routine data collection during our follow-up procedure, information on disease activity and inflammatory markers was not available.

**Table 2:** Indication of maternal JAKi therapy

Substance	Indication	Exposed pregnancies (n)
Baricitinib (*n* = 17)	Rheumatoid arthritis	13
	Atopic dermatitis	2
	Psoriatic arthritis	1
	Alopecia areata	1
Filgotinib (*n* = 3)	Rheumatoid arthritis	2
	Ulcerative colitis	1
Tofacitinib (*n* = 8)	Rheumatoid arthritis	4
	Ulcerative colitis	3
	Juvenile dermatomyositis	1
Upadacitinib (*n* = 16)	Rheumatoid arthritis	8
	Ulcerative colitis	5
	Cohn’s disease	1
	Atopic dermatitis	1
	Spondylarthritis	1

Diagnoses included rheumatological (*n* = 30), gastroenterological (*n* = 10), and dermatological (*n* = 4) diagnoses

### Exposure pattern

**Fig. 2:**
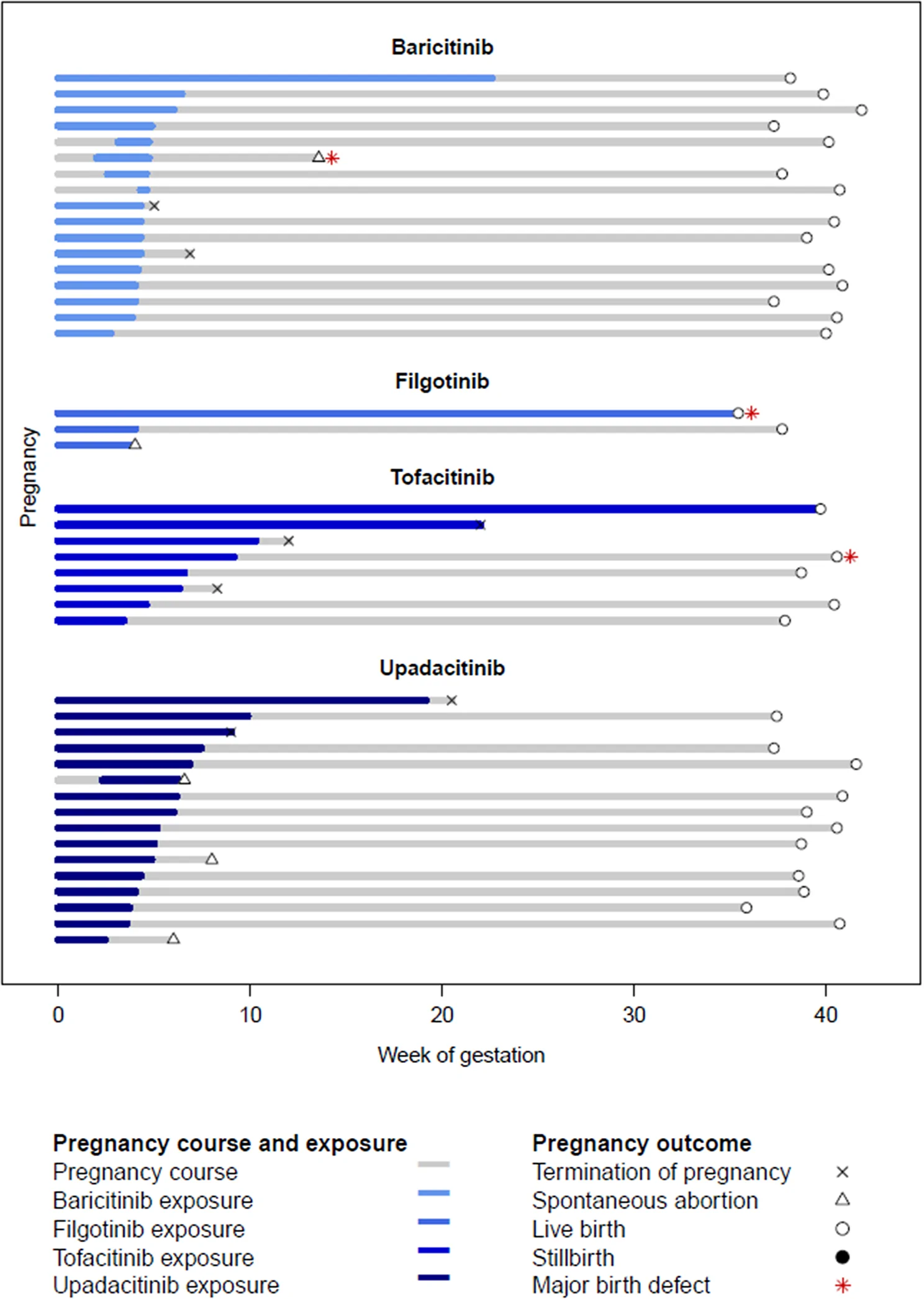
JAK inhibitor exposure and pregnancy outcomes

In the majority of cases, JAKi therapy was discontinued once pregnancy was recognized (Fig. 2). In the subset of patients exposed only during the first trimester (*n* = 39) the median duration of JAKi intake during pregnancy was 4 + 2 gestational weeks (GW, IQR 4 + 0–6+0). Including the five exposures beyond the first trimester, the median GW at discontinuation was 4 + 5 (IQR 4 + 1–6+5).

Co-medication was administered to 25 patients in the first trimester, particular relevant substances included methotrexate exposure (*n* = 2) and systemic glucocorticoids (*n* = 14). After discontinuation of JAKi (*n* = 36), other biological disease modifying drugs (DMARDs) including certolizumab, etanercept, golimumab, adalimumab, and conventional synthetic DMARDs as hydroxychloroquine, mesalamine, sulfasalazine as well as glucocorticoids (prednisolone, budesonide, and triamcinolone) were continued or initiated. In addition, symptomatic treatment involved the use of analgesics (paracetamol, ibuprofen, metamizole) and opium tincture, medical cannabinoids, and loperamide.

### Study endpoints

#### Pregnancy losses

In five pregnancies, spontaneous abortion occurred between GW 4 + 0 and 13 + 4. In one of these, a major anomaly was diagnosed (see below). In seven pregnancies, including one twin pregnancy, an elective termination of pregnancy (ETOP) was reported, all due to personal reasons. Two of the terminations were performed in the advanced second trimester abroad, and not in Germany. No case of stillbirth was reported.

#### Major congenital malformations

Two live-born infants were observed to have major birth defects. One eutrophic full-term female infant was born with a ventricular septal defect (VSD) that did not require surgical intervention. The mother had been diagnosed with rheumatoid arthritis and had been treated with tofacitinib until week 9 + 1 and certolizumab presumably throughout the entire pregnancy. Glandular hypospadias was observed in a male infant born preterm whose mother had been treated with filgotinib for ulcerative colitis and citalopram as a co-medication throughout her pregnancy. Furthermore, anencephaly was diagnosed in one fetus that was miscarried in GW 13 + 4. The pregnant woman being treated for rheumatoid arthritis was exposed to baricitinib, methotrexate, hydroxychloroquine, golimumab, prednisolone, and ibuprofen. The outcomes of pregnancies subsequent to exposure to JAKi are illustrated in Fig. 2.

#### Neonatal characteristics

A total of 32 parities were documented, with all resulting in live-born singletons (Table 3). Of these infants, two were born preterm after 35 + 3 and 35 + 6 weeks of gestation, respectively.

**Table 3:** Neonatal characteristics following intrauterine JAKi exposure (*n* = 32)

Gestational week, n (%)	32 (100)
Gestational week at birth, median week (IQR)	39.4 (37.8–40.6)
**Preterm Birth, n (%)**	32 (100)
Preterm	2 (6.2)
**Child’s sex, n (%)**	32 (100)
Female	13 (40.6)
Male	19 (59.4)
**Weight, n (%)**	32 (100)
Child weight, median g (IQR)	3320 (2910–3540)
**Length, n (%)**	32 (100)
Child length	51.5 (50–53)
**Head circumference, n (%)**	30 (93.8)
Head circumference, median cm (IQR)	34.8 (34–35)

For gestational week at birth, weight, length and head circumference, median and interquartile range (IQR) are presented

The median gestational age and birth weight was 39 + 4 weeks and 3,320 g. Three newborns were found to be small for gestational age, while two were large for gestational age. Further neonatal characteristics can be found in Table 3.

## Discussion

We evaluated a cohort of 44 JAKi exposed pregnancies in the first trimester. Due to the small overall cohort size, limited subgroup sizes for individual JAKi-substances and lack of a comparator group, the data are descriptive and it is not possible to draw definitive conclusions about the teratogenic risks.

In the analyzed study cohort, three major congenital malformations were identified, each affecting different organ systems. A VSD has been reported in a newborn following maternal treatment with tofacitinib up to gestational week 9 + 1. Cardiac septal defects are considered the most common congenital heart anomalies, with a prevalence of approximately 0.4–0.6% among all live-born children [41–44]. To date, one additional case of a septal heart defect has been published in an infant affected by an atrial septal defect (ASD) following maternal exposure to upadacitinib [36].

Furthermore, in the study cohort hypospadias was observed in one child exposed to filgotinib throughout pregnancy. Hypospadias has a general population prevalence of about 0.2–0.8% [42, 45–47]. So far, no further cases of hypospadias have been reported in the literature following JAKi exposure.

Additionally, one fetus in the cohort was affected by anencephaly, resulting in spontaneous abortion. The pregnancy involved complex maternal co-medication, which might have contributed to the adverse outcome. This case report has previously been included in a brief overview of post-marketing safety data [34]. The incidence of anencephaly in the general population is very rare, at around 0.02–0.04% [42, 44, 48].

The current body of evidence for JAKi treatment in pregnancy is limited. All published cohorts have a small cohort size and lack a comparator group, which restricts the ability to conduct robust comparative safety assessments. Most available analyses to date have not identified any alarming patterns of adverse pregnancy outcomes or birth defects. A summary of published studies and case series, including details on reported outcomes by substance, is provided in Table 4. In the literature, following maternal exposure to baricitinib during pregnancy, a total of 26 live births have been reported, including one infant with hip dysplasia [22, 34]. For filgotinib, there are publications on 21 live-born infants following intrauterine exposure, one of whom was diagnosed with pentalogy of Fallot [37]. Tofacitinib exposure during pregnancy has previously been reported in 60 pregnancies resulting in live births; among these, two infants were diagnosed with pulmonary valve stenosis and polydactyly, respectively [20, 21, 27–30, 33, 35]. Additionally, one fetal death at gestational week 32 was reported in a case series in a pregnancy complicated by maternal osteogenesis imperfecta; tofacitinib for the treatment of concurrent alopecia areata was discontinued two weeks after conception [31]. For upadacitinib, one major congenital anomaly of the heart (ASD) was reported among 74 infants exposed to in utero, based on data from the literature [12, 14, 23, 24, 26, 36, 49].

**Table 4:** Summary of published JAKi studies, case series and case reports

Substances	Exposed pregnancies	**1**^**st**^**trimester exposure**	Lost	SAB	ETOP	Still-birth	Live birth	Congenital anomalies	Studies (data cohorts, authors, limitations)
Filgotinib	n = 2	?	0	5	5	0	45	0	Multicenter retrospective study of the ECCO network: Julsgaard et al., 2026 [19], only abstract available, time of exposure unknown, no analysis of outcomes by substance, no comparison cohort
Tofacitinib	n = 38	?							
Upadacitinib	n = 15	?							
Baricitinib	n = 96	At least 65	48	14	9	0	25	2 (anencephaly^1^, hip dysplasia)	Clinical trial and post-marketing sources: Bergamini et al. 2023 [34], sponsored by pharmaceutical industry, high loss rate, time of exposure unknown in many cases, no comparison cohort
	n = 1	1	0	0	0	0	1	0	Case report: Costanzo et al. 2020 [22]
Filgotinib	n = 50	?	10	10	9	0	21	1 (pentalogy of Fallot)	Review of manufacturers’ clinical trial data: Monfared et al. 2023 [37], high loss and ETOP rate, time of exposure unknown, no comparison cohort
Tofacitinib	n = 85	85	12^2^	14	12	0	47	1 (pulmonary valve stenosis^3^)	Retrospective analysis: Mahadevan et al. 2025 [35], sponsored by pharmaceutical industry, high loss and ETOP rate, no comparison cohort
	n = 15	14	0	0	1	1^4^	13	1 (preaxial polydactyly [21])	Case reports and series: Arzivian et al., 2024 [27]; Chaparro et al., 2024 [21]; Ernest-Suarez et al., 2024 [28]; Fernandez-Sanchez et al., 2021 [29]; Mitrova et al., 2025 [20, 30]; Ogbutor et al., 2025 [31]; Rowan et al., 2024 [33]; Zhang et al., 2024 [32]
Upadacitinib	n = 128	At least 112	0	37	26	0	65	1 (atrial septal defect)	Clinical trial and postmarketing cases: Mahadevan et al. 2024 [36], sponsored by pharmaceutical industry, high SAB and ETOP rate, time of exposure unknown in some cases, no comparison cohort
	n = 9	5	0	0	0	0	9	0	Case reports and series: Coman et al., 2025 [23]; Duricova et al., 2025 [14]; Ehrlich et al., 2025 [12]; Gargiulo et al., 2023 [25]; Geeganage et al., 2025 [26]; Urquhart et al., 2025 [24]

Abbreviations: ECCO, European Crohn’s and Colitis Organisation; ETOP, elective termination of pregnancy; SAB, spontaneous abortion

^1^ According to the case description it is the same case as in the Embryotox study cohort (co-medication: methotrexate, golimumab, hydroxychloroquine); ^2^ including one fetal death caused by maternal lethal accident; ^3^ co-medication: angiotensine-2-receptor antagonist; ^4^ one stillbirth 28 weeks after last exposure, presumably associated with maternal osteogenesis imperfecta

In the cohort analyzed here, five spontaneous abortions were observed among the 44 pregnancies with first-trimester exposure to JAKi. Given that the medium time of first contact with Embryotox was 6 + 3 weeks of gestation, data on miscarriages occurring earlier in pregnancy are not available. However, the observed number of spontaneous abortions in this small cohort does not appear to be elevated and is consistent with the expected background rate after clinical recognition of pregnancy, which is about 12.5–18.7% [50, 51]. The high number of elective terminations of pregnancy (seven out of 44 pregnancies) in the study cohort is noteworthy. While some terminations might reflect general ambivalence toward pregnancy within the cohort (see Table 1), others could be influenced by concerns regarding a potential teratogenicity of JAKi. This observation has previously been reported in analyses of manufacturer-sponsored registry data [34–36]. As a result, one could speculate that adverse fetal outcomes, such as congenital anomalies may have been underreported or missed in pregnancies that ended in ETOP or spontaneous abortions.

Neonatal characteristics were unremarkable in the analyzed study cohort. Birth weights were within the expected normal range, as well as the observed number of SGA and LGA infants [52].

In 2025, a pharmacovigilance disproportionality analysis was conducted in VigiBase, the World Health Organization global database for adverse event reports for medicines and vaccines. Signals of disproportionate reporting (SDRs) were evaluated for pregnancy-related adverse drug reactions associated with 13 different JAKi. The analysis included 163 individual case safety reports (ICSRs) from pregnant individuals, and tofacitinib (44.8%) and upadacitinib (34.4%) were the most frequently reported JAKi in the cohort. No significant SDRs were identified for major congenital anomalies, spontaneous abortion, or prematurity. Furthermore, no consistent or specific pattern of major congenital anomalies was observed [53].

In the analyzed study cohort, JAKi exposure was heterogeneous. Only three live-born infants were exposed to JAKi throughout the entire first trimester. Consequently, the findings of this case series cannot be generalized to situations involving continuous exposure during first trimester or throughout pregnancy.

Moreover, due to the broad spectrum of rheumatological, gastroenterological, and dermatological treatment indications of underlying conditions, along with the exposure to four different JAKi in the present cohort, it remains uncertain whether any observed pregnancy outcomes are attributable to the underlying diseases, the pharmacological effects of the drugs, a combination of both, or other factors. The heterogeneity in disease severity, treatment duration, and concomitant medication use further complicates the interpretation of potential drug-related risks. As such, disentangling the impact of JAKi poses a significant challenge.

Another limitation of this study is its reliance on a voluntary reporting procedure, which carries an inherent risk of selection and reporting bias [54]. Previous analyses have shown that women enrolled in the Embryotox program tend to be more educated, which may skew the cohort toward individuals with better access to healthcare and greater awareness of reproductive risks [55]. This could potentially lead to underreporting of adverse outcomes in less-educated or medically underserved populations.

Despite these limitations, the study cohort benefits from a prospective, standardized, and systematically structured data collection process. All pregnancies were documented using predefined criteria and validated clinical assessments, which enhances the reliability and consistency of the collected information [56].

In summary, three major congenital anomalies were reported in a cohort of 44 individuals. No specific pattern was identified, as different organ systems were affected. The number of spontaneous pregnancy loss was not suspicious. However, due to the aforementioned limitations in study design and the inherent challenges in assessing drug safety during pregnancy, the results should be interpreted with caution and alongside other published knowledge.

## Conclusions

The available data are insufficient to finally assess embryotoxicity of JAKi in humans. Consequently, the use of JAKi during pregnancy should be restricted to individualized clinical decision-making, and considered only in situations of high disease activity where no established and effective alternative therapies are available. There is an urgent need for additional research, especially considering the growing clinical use of these agents among reproductive-age individuals.

## Data Availability

The anonymous datasets analyzed for the current study are available from the corresponding author on reasonable request.

## Abbreviations

ASD: Atrial septal defect
BfArM: Bundesinstitut für Arzneimittel und Medizinprodukte (Federal Institute for Drugs and Medical Devices)
BMI: Body mass index
DMARDs: Disease-modifying antirheumatic drugs
ECCO: European Crohn’s and Colitis Organisation
EDOB: Estimated date of birth
ETOP: Elective termination of pregnancy
EUROCAT: European network of population-based registries for the epidemiological surveillance of congenital anomalies
GW: Gestational week
ICSR: Individual case safety reports
IQR: Interquartile range
JAK: Janus kinase
JAKi: Janus kinase inhibitors
JAK/STAT: Janus kinase signal transducer and activator of transcription
LGA: Large for gestational age
LMP: Last menstrual period
SAB: Spontaneous abortion
SDR: Signals of disproportionate reporting
SGA: Small for gestational age
TYK: Tyrosine kinase
VSD: Ventricular septal defect
